# Molecular assembly indices of mineral heteropolyanions: some abiotic molecules are as complex as large biomolecules

**DOI:** 10.1098/rsif.2023.0632

**Published:** 2024-02-21

**Authors:** Robert M. Hazen, Peter C. Burns, H. James Cleaves, Robert T. Downs, Sergey V. Krivovichev, Michael L. Wong

**Affiliations:** ^1^ Earth and Planets Laboratory, Carnegie Institution for Science, Washington, DC 20015, USA; ^2^ Department of Civil and Environmental Engineering and Earth Sciences, University of Notre Dame, Notre Dame, IN 46556, USA; ^3^ Department of Chemistry and Biochemistry, University of Notre Dame, Notre Dame, IN 46556, USA; ^4^ Earth Life Science Institute, Tokyo Institute of Technology, Tokyo 152-8550, Japan; ^5^ Blue Marble Space Institute for Science, Seattle, WA 98104, USA; ^6^ Department of Chemistry, Howard University, Washington, DC 20059, USA; ^7^ Geological Sciences, University of Arizona, Tucson, AZ 85721, USA; ^8^ Department of Crystallography, Institute of Earth Sciences, St. Petersburg State University, St. Petersburg 199034, Russia; ^9^ Nanomaterials Research Centre, Kola Science Centre, Russian Academy of Sciences, Fersmma 14, Apatity 184209, Russia; ^10^ Sagan Fellow, NASA Hubble Fellowship Program, Space Telescope Science Institute, Baltimore, MD 21218, USA

**Keywords:** assembly theory, molecular complexity, heteropolyanion, mineral evolution, Titan

## Abstract

Molecular assembly indices, which measure the number of unique sequential steps theoretically required to construct a three-dimensional molecule from its constituent atomic bonds, have been proposed as potential biosignatures. A central hypothesis of assembly theory is that any molecule with an assembly index ≥15 found in significant local concentrations represents an unambiguous sign of life. We show that abiotic molecule-like heteropolyanions, which assemble in aqueous solution as precursors to some mineral crystals, range in molecular assembly indices from 2 for H_2_CO_3_ or Si(OH)_4_ groups to as large as 21 for the most complex known molecule-like subunits in the rare minerals ewingite and ilmajokite. Therefore, values of molecular assembly indices ≥15 do not represent unambiguous biosignatures.

## 1. Introduction

Cronin, Walker, and colleagues [1–8] have championed the assembly theory (AT) approach to characterizing complex evolving systems. In their framework, evolving systems are made from objects (e.g. molecules; automobiles) that must be assembled through a series of steps from smaller building units (e.g. chemical bonds; automobile parts). Furthermore, assembly can be imagined to occur through a minimum number of discrete steps called the ‘assembly index’. In the AT model, the products of living systems, whether biomolecules or automobiles, are distinguished from nonliving objects by two coexisting criteria: (1) the object requires numerous assembly steps and (2) the object is present in numerous copies—in ‘high abundance’. Only a directed assembly by life can overcome the combinatorial improbability of objects as complex as ribosomes or Bentleys occurring multiple times.

AT has not gone unchallenged. Some reviewers question whether a scalar assembly index can be employed to adequately discriminate between living and nonliving systems [9,10]. Other critics note similarities of the AT approach to uncited prior efforts to distinguish biotic from abiotic molecular suites by statistical or algorithmic measures [11]. In particular, Hernández-Orozco and colleagues [12] may have anticipated key conclusions of assembly theory by exploring connections among causal memory, selection, and evolution. This hypothesis has also received criticism based on a variety of concerns, including ambiguities in the numbers of molecular copies that constitute ‘high abundance’, the optimal algorithm to calculate ‘pathway complexity’, the disconnect between the proposed theoretical assembly pathways and actual chemical processes, and the absence of kinetic and thermodynamic factors in assessing the probability for a complex molecule's formation [9,10,13].

In the context of their ambitious research programme, the AT group has developed molecular assembly (MA) indices to characterize the number of unique chemical bonding steps necessary to build discrete molecules from their constituent atoms. Such indices range from 1 for the simplest molecules, to more than 15 for many molecules in cells, to greater than 30 for the most complex biomolecules [2–7]. Cronin, Walker, and colleagues conclude that any organic molecule that occurs abundantly (defined variously as being present in greater than a million copies or producing measurable peaks in mass spectrometric analysis) and that has MA index ≥15 is a uniquely biological signature. Such complex molecules, they explain, represent combinatoric rarities that are too unlikely to be repeatedly synthesized without highly controlled cellular processes. They extend this idea to postulate that only living systems can produce molecular structures of such complexity.

Here we test this quantitative AT hypothesis by examining some of the most complex abiotic molecule-like heteropolyanions (also known as polyoxometalates in oxide minerals) that are building blocks of inorganic mineral structures to test the intriguing conjecture that ‘One thing that discriminates living things from inanimate matter is their ability to generate similarly complex or non-random structures in a large abundance’ [1]. Specifically, Marshall *et al*. [3] propose that there exists a ‘threshold abiotic-biotic divide, … which can be used to unambiguously assign complex objects as biosignatures’. In this context, structural complexity is measured by ‘pathway complexity’ or ‘molecular assembly index’—a numerical value that is defined by the number of unique steps required ‘to assemble a given object by allowing the object to be dissected into a set of basic building units and rebuilding the object using those units’.

A complication arises from the use of two similar yet differing published protocols for calculating MA indices. One from Marshall *et al*. [2–4], called the ‘split-branch algorithm’, is simpler to implement and provides an ‘upper bound’ on the MA index of a molecule. The other more rigorous approach (‘exact MA’, which can be derived via Monte Carlo methods) is described by Cronin, Walker, and colleagues [5,6], who provide a computer program to calculate minimum MA indices [7]. In both instances, the calculation of MA indices relies on the sequential formation of links between ‘basic building units’, which are defined as chemical bonds between two atoms other than hydrogen. Typical basic building units in organic molecules include (C–C), (C=C), (C=O), and (C–N).

In the case of the split-branch algorithm [2, fig. 2A] (see also [4]), each bond added to an initial unit is counted as a MA step. Thus, the initial step is always formation of a bond, such as C=C, followed by attachment of additional bonds. Therefore, the assemblies of (C=C) + (C–C) → (C=C–C) in tryptophan or (N=C) + (C–N) → (N=C–N) in ATP require 2 assembly steps. Similarly, in the split-branch version of MA index calculations the assemblies of a (C_3_N) group in asparagine or (CO_3_) carbonate group, each with a central carbon atom linked to 3 other atoms, requires 3 assembly steps. The resulting assembly indices for asparagine and tryptophan are 7 and 12, respectively [2].

**Figure 1.**
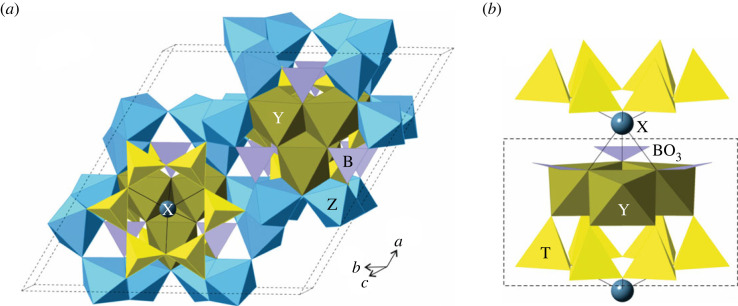
The crystal structure of the tourmaline group mineral fluor-uvite [14] projected approximately parallel to the *c* axis (*a*) and the (Mg_3_B_3_Si_6_O_36_)^33−^ island (*b*). In fluor-uvite: *X* = Ca, *Y* = Mg, *Z* = Al, *T* = Si.

Alternatively, in the exact MA calculation protocol (e.g. [5, fig. 1]; see also [7]), the initial formation of a bond is not counted as a step; the MA index depends entirely on the number of ‘joining operations’. Thus, forming (C=C–C) in tryptophan or (N=C–N) in ATP requires only 1 assembly step, for example to join (C=C) with (C–C), while assembly of a (C_3_N) group in asparagine or (CO_3_) carbonate group requires only 2 steps. Furthermore, assembly of (SiO_4_) requires only 2 steps in the exact MA algorithm: (Si–O) × 2 → (SiO_2_); then (SiO_2_) × 2 → (SiO_4_). Employing the exact MA method, the resulting assembly indices for asparagine and tryptophan are reduced from 7 and 12 [2] to 6 and 11, respectively [7], demonstrating that the split-branch index is usually greater than exact MA index [4,7].

In the exact MA approach [5–7], the assembly index of any molecule begins with an already formed bond; no initial step is required to join two non-H atoms (e.g. ethane (H_3_CCH_3_) or carbon monoxide (CO)). Consequently, the assembly index for these small molecules is 0, whereas a variety of tri-atomic molecules, including (CO_2_) and (HCN), and C_3_ hydrocarbons (propane, propene) have exact assembly indices of 1 [5–7]. Addition of other building units leads to progressively greater MA indices, for example 3 for cyclopentane (C_5_H_10_), 4 for glycine (H_2_NCH_2_COOH), and 5 for alanine (H_2_NCH(CH_3_)COOH).

For larger organic molecules, which often incorporate a combination of chains, branches, and/or rings in an open configuration, assembly may proceed from one region of the molecule to the next by the addition of new bonds or small molecular subunits one at a time. Once a subunit has been synthesized, that unit can be used repeatedly in the subsequent assembly process without requiring additional steps. Cronin and colleagues [5,6] provide several examples of exact MA index calculations up to MA index >15, as well as a program to calculate exact MA indices for a wide range of organic molecular species [7].

A difficulty remains. While exact MA index calculations are presented in [5–7], the proposed abiotic/biotic boundary of MA index ≥15 was based on the split-branch MA indices of Marshall *et al*. [2]. Because the Marshall *et al*. method usually overestimates MA indices by at least 1 or 2 steps, it seems probable that the abiotic/biotic boundary for exact MA indices may be 13 or 14, rather than ≥15. Because of this ambiguity, in the following section we calculate both the split-branch and exact assembly indices of molecule-like mineral substructures.

Mineral structures, in contrast to most organic molecules, tend to be dense arrangements of repeating small atom clusters (i.e. cation polyhedra such as (SiO_4_) tetrahedra or (MgO_6_) octahedra) that may share corners, edges, and/or faces with adjacent polyhedra. Thousands of three-dimensional atomic arrangements in minerals have been documented [15], some of which are exceedingly complex with as many as a dozen essential elements and hundreds of symmetrically distinct atom sites in the asymmetric unit. Many pathways will exist to assemble any such three-dimensional structure starting with individual atoms, though an assembly pathway of shortest length must exist. Nevertheless, according to the conventions of assembly theory, which only relates to monodispersed and countable copies of a molecule, neither mineral crystals nor polymers are considered no matter how complex the structures might be [8]. Therefore, in this contribution we consider only polyatomic inorganic clusters that are assumed to be aqueous precursors to minerals that directly incorporate such substructures from solution [16–18]. These minerals feature clusters of atoms in which cations of oxidation states 2+ or greater form well-defined substructures that are linked internally by relatively strong bonds. By contrast, adjacent clusters link to each other through relatively weak bonds, such as [(Na,K)-O] or hydrogen bonds.

**Table:** 

Mineralogical glossary	
*asymmetric unit*	the fraction of the unit cell that, when transformed by symmetry operators, generates the complete unit cell. The asymmetric unit of minerals contain from one to more than 200 distinct atomic sites
*essential element*	an essential element is a chemical element that is required by the International Mineralogical Association's (IMA's) definition of the mineral species
*heteropolyanion*	a strongly bonded cluster of atoms with two or more kinds of cations in relatively high oxidation state bonded to oxygen atoms; a synonym of polyoxometalates
*polyhedron*	a small atom cluster with a central cation or anion surrounded by several nearest neighbour atoms of the opposite charge, e.g. (SiO_4_)^4−^
*polyoxometalates*	polyoxometalates are nanoscale metal-oxide clusters that spontaneously assemble in aqueous solution; a synonym of heteropolyanion
*mineral species*	one of approximately 6000 mineral kinds approved by the IMA's Commission on New Minerals, Nomenclature, and Classification. Each species has a unique combination of idealized composition and crystal structure
*solid solution*	a solid solution occurs when two or more elements occupy the same crystallographic site in varying ratios. Common examples of solid solution include (Mg-Fe^2+^), (Al-Fe^3+^), and (OH-F)
*unit cell*	the parallelepiped-shaped volume that repeats in the crystal structure
*Wyckoff site*	a crystallographically unique site in the unit cell

## 2. Molecular assembly indices for selected mineral heteropolyanions

The International Mineralogical Association's Commission on New Minerals, Nomenclature, and Classification (IMA-CNMNC) recognizes approximately 6000 different mineral ‘species’, each defined by its unique combination of idealized chemistry and crystal structure [15]. The majority of these mineral structures are ‘space-filling’ arrangements of atoms, and thus do not feature distinct molecule-like regions amenable to assembly index calculations. Well-known examples include such high-symmetry phases as native iron (Fe), halite (NaCl), and spinel (MgAl_2_O_4_). Other minerals, most notably silicates such as quartz (SiO_2_), alkali feldspar [(Na,K)AlSi_3_O_8_], and scores of framework zeolite group phases, are highly polymerized three-dimensional arrays of corner-linked [(Si,Al)O_4_] tetrahedra. According to conventions of molecular assembly theory, these repeating three-dimensional structures have undefined molecular assembly indices [8]. Therefore, we focus exclusively on minerals that incorporate convincing molecule-like subunits that are thought to form from aqueous precursors.

Scores of complex mineral structures feature strongly bonded polyatomic subunits that are molecular in character, that likely assemble in an aqueous or silicate melt environment prior to crystallization, and for which a molecular assembly index can be calculated unambiguously. In some cases, direct evidence exists supporting a mechanism of molecular assembly in solution prior to crystallization [16–18]. In table 1, we provide examples of minerals with such molecule-like subunits with calculated assembly indices for those subunits based both on the split-branch protocols of Marshall *et al*. [2–4] and the more recent ‘exact’ protocols [5–7]. We also list the chemical metric of the number of different essential chemical elements—i.e. an element that is required in the IMA's definition of the mineral species, as well as a structural complexity metric (see below).

**Table 1.** Complexity metrics for molecule-like subunits of select minerals, including estimated split-branch (SBMA) and exact (EMA) indices.

mineral	formula	molecular subunit	SBMA	EMA	no. of elements^a^	Kriv info^b^
calcite	Ca(CO_3_)	(CO_3_)^2−^	3	2	3	13.7
forsterite	Mg_2_(SiO_4_)	(SiO_4_)^4−^	3	2	3	70.6
åkermanite	Ca_2_Mg(Si_2_O_7_)	(Si_2_O_7_)^6−^	5	3	4	58.0
beryl	Be_3_Al_2_(Si_6_O_18_)	(Si_6_O_18_)^12−^	6	5	4	120
vesuvianite	(Ca,Na)_19_(Al,Mg,Fe)_13_(SiO_4_)_10_(Si_2_O_7_)_4_(OH,F,O)_10_	[(SiO_4_)(Si_2_O_7_)(AlO_4_)]^15−^	10	8	9	1229
fluor-uvite	CaMg_3_(Al_5_Mg)(Si_6_O_18_)(BO_3_)_3_(OH)_3_F	(Mg_3_B_3_Si_6_O_36_)^33−^	14	11	7	200
eudialyte	Na_15_Ca_6_Fe_3_Zr_3_Si(Si_25_O_73_)(O,OH,H_2_O)_3_(Cl,OH)_2_	[Ca_6_(Si_3_O_9_)_2_(Si_3_O_10_)_6_]^30−^	12	10	8	529
		[(Si_9_O_27_)_2_(ZrO_4_)_6_]^60−^	12	10		529
vanarsite	NaCa_12_(As^3+^V^4+^_2_V^5+^_10_As^5+^_6_O_51_)_2_·78H_2_O	[(As^3+^V^4+,5+^_12_As^5+^_6_)O_51_]^32−^	16	11	6	5990
paddlewheelite	MgCa_5_Cu_2_(UO_2_)_4_(CO_3_)_12_(H_2_O)_33_	[Cu^2+^Ca^2+^_2_U^6+^_4_O_12_(CO_3_)_12_]^18−^	17	13	7	5876
ewingite	Mg_8_Ca_8_(UO_2_)_24_(CO_3_)_30_O_4_(OH)_12_(H_2_O)_138_	[(U^6+^O_2_)_24_(CO_3_)_30_O_4_]^20−^	20	18	6	23 478
ilmajokite	Na_11_KBaCe_2_Ti_12_Si_37.5_O_94_(OH)_30_·29H_2_O	[Ce(Ti_2_SiO_12_)_3_(Si_17_O_35_)]^35−^	21	18	8	11 990

^a^The number of essential elements in the mineral's chemical formula.

^b^Calculations of mineral structural complexity are detailed in [19–21].

**Figure 2.**
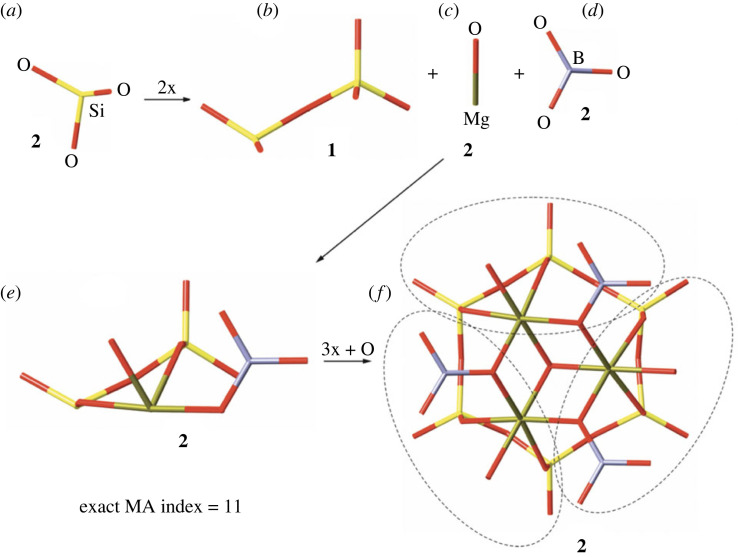
The ‘exact’ MA index assembly pathway for the fluor-uvite cluster shown in figure 1*b*: the basic element of the silicate ring (*a*); three basic elements that include dimeric silicate unit (*b*), MgO bond (*c*) and BO_3_ triangle (*d*); the linkage of the three basic elements shown in (*b*) into a complex unit (*e*); the resulting fluor-uvite island centred by additional anion (*f*; the outlines of the subunits shown in (*e*) are indicated by the dashed lines). The value of MA index is written below each structure.

The simplest ‘molecules’ in minerals are polyhedra of high-field-strength cations (typically oxidation state 3+ or greater) bonded to oxygen atoms. These rigid molecule-like subunits are linked by 1+ or 2+ cations and/or hydrogen bonds in interstitial regions. Common examples of simple, rigid polyhedra include the (CO_3_)^2−^ carbonate group, the (BO_3_)^3−^ borate group, the (SiO_4_)^4−^ orthosilicate group, and the (SO_4_)^2−^ sulfate group. In all cases, we calculate assembly indices with bonds between two non-hydrogen atoms as building blocks; however, the MA indices differ depending on which of two published protocols are employed. Using the Marshall *et al*. split-branch procedures [2, fig. 2*a*], (CO_3_) and (SiO_4_) each require 3 assembly steps. Alternatively, in the Liu *et al*. [4] exact approach both (CO_3_) and (SiO_2_) require only 2 assembly steps. In the case of (CO_3_), first combine two (C–O) bonds to form (CO_2_); then add a third (C–O) bond. Similarly, in the exact MA approach the formation of (SiO_4_) requires 2 steps: 2 (Si–O) bonds form (SiO_2_); then 2 (SiO_2_) groups combine into (SiO_4_). It is important to note that *the bonds, not the atoms, are combined* in these operations.

Silicate tetrahedra often link together to form larger clusters, including in various disilicates (Si_2_O_7_)^6−^ and cyclosilicates with three-member (Si_3_O_9_)^6−^ and six-member (Si_6_O_18_)^12−^ rings. In the split-branch approach these molecular subunits have assembly indices of 5, 5, and 6, respectively. Alternatively, in the exact approach the indices are 4, 4, and 5, respectively.

Additional complexity arises when silicate clusters combine with other elements. In beryl [Be_3_Al_2_Si_6_O_18_], for example, 6-member (Si_6_O_18_)^12−^ rings are cross-linked by six corner-sharing AlO_6_ octahedra to form a three-dimensional framework. Several examples will clarify molecular assembly calculations.

### 2.1. Vesuvianite

Among the most common minerals incorporating disilicate groups is the hydrous calcium-aluminium silicate vesuvianite [(Ca,Na)_19_(Al,Mg,Fe)_13_(SiO_4_)_10_(Si_2_O_7_)_4_(OH,F,O)_10_], which occurs abundantly in Ca-rich metamorphic rocks [15]. A molecule-like [(SiO_4_)(Si_2_O_7_)(AlO_4_)]^15−^ subunit of vesuvianite exemplifies the steps of a molecular assembly using both split-branch (SBMA) and exact (EMA) approaches:

**Table:** 

(Si–O) × 2 → (SiO_2_)	SBMA = 2 steps	EMA = 1 step
(Si–O) + (SiO_2_) → (SiO_3_)	1 step	1 step
(SiO_2_) × 2 → (SiO_4_)	1 step	1 step
(SiO_4_) + (SiO_3_) → (Si_2_O_7_)	1 step	1 step

Silicate groups bond via corner sharing (AlO_6_)^9−^ octahedra:

**Table:** 

(Al–O) × 2 → (AlO_2_)	2 steps	1 step
(AlO_2_) × 2 → (AlO_4_)	1 step	1 step
(AlO_4_) + (SiO_4_) → [(SiO_4_)(AlO_4_)]	1 step	1 step
[(SiO_4_)(AlO_4_)] + (Si_2_O_7_) → [(SiO_4_)(Si_2_O_7_)(AlO_4_)]	1 step	1 ste
TOTAL	SBMA = 10 steps	EMA = 8 steps

Thus, the assembly index of this [(SiO_4_)(Si_2_O_7_)(AlO_4_)]^15−^ molecule-like subunit is either 8 or 10 steps, depending on the MA index calculation method used—values significantly below the abiotic/biotic divide of Marshall *et al*. [3]. In the following sections, we consider six more complex structures containing molecule-like subunits with MA index ≥ 10.

### 2.2. Tourmaline group

Cyclosilicate structures often feature molecule-like subunits that extend beyond the silicate ring. The varied tourmaline group, with at least 38 species that form in both igneous and metamorphic environments [15], has a generalized formula of *XY*_3_*Z*_6_*T*_6_O_18_(BO_3_)_3_*V*_3_*W*, with typical constituent elements including *X* = Na, K, or Ca; *Y* and *Z* = Al, Cr, V, Fe, Mg; *T* = Si, Al; *V* = (OH), O; and *W* = (OH), F, O. Note that the substitution of multiple elements in a given site via ‘solid solution’, while increasing a specimen's chemical complexity, does not increase the assembly index. In the species fluor-uvite [CaMg_3_(Al_5_Mg)(Si_6_O_18_)(BO_3_)_3_(OH)_3_F], for example, one can consider a ‘structural island’ [14] consisting of a (Si_6_O_18_)^12−^ ring bonded to a group of three edge-sharing MgO_6_ octahedra in a (Mg_3_O_12_)^18−^ cluster and three planar (BO_3_)^3−^ molecular subunits (figure 1). This molecule-like group with composition (Mg_3_B_3_Si_6_O_36_)^33−^ can be constructed in a minimum of 11 steps (figure 2). In this example, the minimum assembly pathway is not immediately obvious. Rather than first assemble the (Si_6_O_18_)^12−^ ring and expand outward, the optimal path involves assembly of a (MgBSi_2_O_12_)^11−^ subunit that can be repeated by tourmaline's trigonal symmetry:

**Table:** 

(Si–O) × 3 → (SiO_3_)	SBMA = 3 steps	EMA = 2 steps
(SiO_3_) × 2 → (Si_2_O_6_)	1 step	1 step
(Mg–O) × 3 → (MgO_3_)	3 steps	2 steps
(Si_2_O_6_) + (MgO_3_) → (MgSi_2_O_9_)	1 step	1 step
(B–O) × 3 → (BO_3_)	3 steps	2 steps
(MgSi_2_O_9_) + (BO_3_) → (MgBSi_2_O_12_)	1 step	1 step

At this point, three identical (MgBSi_2_O_12_) subunits can be linked in 2 steps:

**Table:** 

(MgBSi_2_O_12_) × 3 → (Mg_3_B_3_Si_6_O_36_)	2 steps	2 steps
TOTAL	SBMA = 14 steps	EMA = 11 steps

The assembly index for the tourmaline cluster is thus 14 or 11, depending on the method used. Note that this cluster as written has a large negative charge (−33 in this case)—a situation typical of many other molecular clusters in minerals. In an aqueous environment, this charge would be compensated by the formation of numerous peripheral (O–H) bonds, which are not considered in the calculation of assembly indices. Alternatively, the addition of apical F atoms instead of OH^−^, as is common in tourmaline (thus forming (Mg–F) or (Si–F) bonds as part of the cluster) might increase the exact MA index to 12 or greater.

In this example, tourmaline displays a characteristic shared by many high-symmetry clusters. In spite of the fact that the cluster contains more than 100 cation-oxygen bonds, the assembly index is only 11 or 14 for exact or split-branch calculations, respectively, as a consequence of the repeated use of subunits such as (MgSi_2_O_9_) and (BeO_3_), which only need to be assembled once.

### 2.3. Eudialyte group

**Figure 3.**
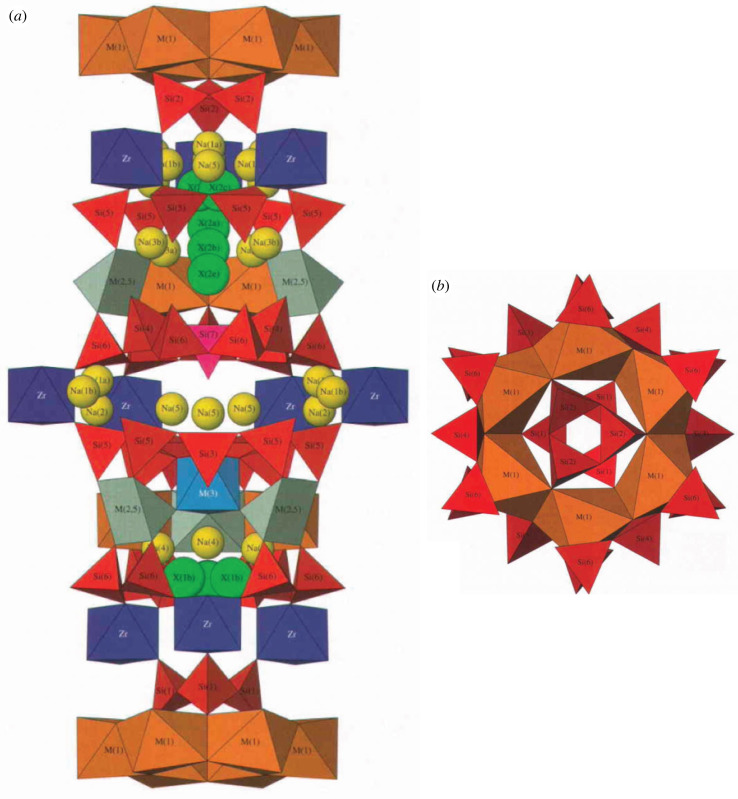
(*a*) The eudialyte [Na_15_Ca_6_Fe_3_Zr_3_Si(Si_25_O_73_)(O,OH,H_2_O)_3_(Cl,OH)_2_] structure [22] features sodium atoms (yellow spheres), Ca polyhedra (orange), Fe polyhedra (grey), Zr octahedra (dark blue), Si–Nb polyhedra (light blue), Si tetrahedra (red), and Cl atoms (green spheres). Hydrogen atoms are not shown. (*b*) Eudialyte incorporates at least two different molecule-like sub-assemblies. One example, shown here, includes an edge-sharing six-member ring of calcium octahedra (in orange), with three-member (Si_3_O_9_) rings (red) above and below and six additional (Si_3_O_10_) three-tetrahedron segments around the periphery to form a [Ca_6_(Si_3_O_9_)_2_(Si_3_O_10_)_6_]^30−^ cluster. We also focus on the strongly bonded sandwich-like subunit of (ZrO_6_) octahedra between two (Si_9_O_27_) rings [(Si_9_O_27_)_2_(ZrO_4_)_6_]^60−^, as represented by adjacent red and dark blue polyhedra in (*a*). (From Johnson & Grice [22], used with permission from the *Canadian Journal of Mineralogy and Petrology*.)

Complex molecular subunits often arise when silicate structures are coupled with other high-field-strength cations, such as V^4+^, V^5+^, As^5+^, Zr^4+^, and/or U^6+^ into structures called heteropolyanions. A common example is provided by the diverse eudialyte group (figure 3*a*), with more than 30 species that form exclusively in high-temperature igneous environments associated with alkali-rich, silica-poor magmas [15,22,23]. A typical species is the eponymous eudialyte [Na_15_Ca_6_Fe_3_Zr_3_Si(Si_25_O_73_)(O,OH,H_2_O)_3_(Cl,OH)_2_]. Eudialyte incorporates at least two different molecule-like sub-assemblies. One example (figure 3*b*) includes an edge-sharing six-member ring of calcium octahedra (shown in orange in figure 3*b*), with three-member (Si_3_O_9_) rings (red) above and below and six additional (Si_3_O_10_) three-tetrahedron segments around the periphery to form a [Ca_6_(Si_3_O_9_)_2_(Si_3_O_10_)_6_]^30−^ cluster. Here, again, the optimal strategy for calculating a minimum assembly pathway is to exploit this cluster's 6-fold symmetry by first assembling a [Ca(SiO_3_)(Si_3_O_10_)]^6−^ subunit. Start by considering the silicate groups:

**Table:** 

(Si–O) × 3 → (SiO_3_)	SBMA = 3 steps	EMA = 2 steps
(SiO_3_) × 2 → (Si_2_O_6_)	1 step	1 step
(SiO_3_) + (Si–O) → (SiO_4_)	1 step	1 step
(Si_2_O_6_) + (SiO_4_) → (Si_3_O_10_) [3 tetrahedra chain]	1 step	1 step

These silicate groups are linked by the calcium atom:

**Table:** 

(SiO_3_) + (Ca–O) → [Ca(SiO_3_)]	2 steps	1 step
[Ca(SiO_3_)] + (Si_3_O_10_) → [Ca(SiO_3_)(Si_3_O_10_)]	1 step	1 step
[Ca(SiO_3_)(Si_3_O_10_)] × 2 → [Ca_2_(SiO_3_)_2_(Si_3_O_10_)_2_]	1 step	1 step
[Ca_2_(SiO_3_)_2_(Si_3_O_10_)_2_] × 3 → [Ca_6_(Si_3_O_9_)_2_(Si_3_O_10_)_6_]	2 steps	2 steps
TOTAL	SBMA = 12 steps	EMA = 10 steps

Thus, the assembly index is 10 or 12, depending on the method used. Note that the final assembly steps involve the simultaneous formation of 6 or more bonds between two subunits—a situation typical for assembly of high-symmetry clusters. As in the previous examples, H atoms must decorate the apical oxygens of this cluster in solution to maintain a low net molecular charge; nevertheless, O–H bonds are not included in the assembly calculation.

Another key molecular subunit of eudialyte is [(Si_9_O_27_)_2_(ZrO_4_)_6_]^60−^, which consists of a sandwich of 9-member (Si_9_O_27_)^18−^ silicate rings above and below, linked by six (ZrO_6_)^6−^ octahedra (figure 3*a*). In this case, the first steps involve assembling a [(Si_3_O_9_)_2_(ZrO_4_)_2_]^20−^ subunit, thus exploiting the cluster's 3-fold symmetry. Start with assembly of the (Si_3_O_9_) and (ZrO_4_) polyhedral units:

**Table:** 

(Zr–O) × 2 → (ZrO_2_)	SBMA = 2 steps	EMA = 1 step
(ZrO_2_) × 2 → (ZrO_4_)	1 step	1 step
(Si–O) × 3 → (SiO_3_)	3 steps	2 steps
(SiO_3_) × 3 → (Si_3_O_9_)	2 steps	2 steps

Combine those units in the [(Si_3_O_9_)_2_(ZrO_4_)_2_] subassembly:

**Table:** 

(Si_3_O_9_) + (ZrO_4_) → [(Si_3_O_9_)(ZrO_4_)]	1 step	1 step
[(Si_3_O_9_)(ZrO_4_)] × 2 → [(Si_3_O_9_)_2_(ZrO_4_)_2_]	1 step	1 step

Finally, combine 3 subassemblies into the cluster:

**Table:** 

[(Si_3_O_9_)_2_(ZrO_4_)_2_] × 3 → [(Si_9_O_27_)_2_(ZrO_4_)_6_]	2 steps	2 steps
TOTAL	SBMA = 12 steps	EMA = 10 steps

Thus, the molecular assembly of this eudialyte subunit is 10 or 12, depending on the method used. Note that adding on to this subunit are sodium, calcium, and iron atoms, as well as three-member (Si_3_O_10_)^18−^ molecules, thus significantly increasing the structural complexity of eudialyte.

### 2.4. Vanarsite and related structures

**Figure 4.**
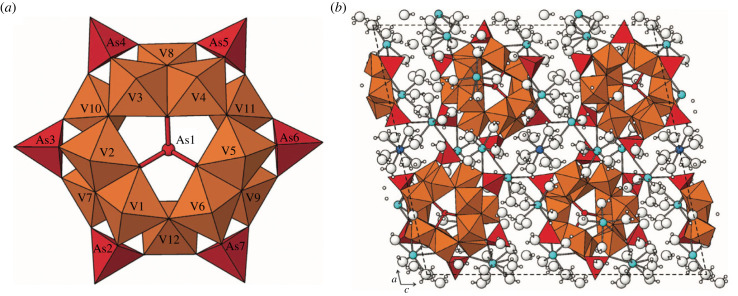
(*a*) The [(As^3+^V^4+,5+^_12_As^5+^_6_)O_51_] heteropolyanionic cluster found in several mineral species is a strongly bonded subunit of a more extensive structure. Arsenic polyhedra are shown in red, while vanadium polyhedra are shown in orange [24]. (*b*) The crystal structure of vanarsite [NaCa_12_(As^3+^V^4+^_2_V^5+^_10_As^5+^_6_O_51_)_2_·78H_2_O] reveals weakly bonded interstitial regions with Na–O, Ca–O, and hydrogen bonding between polyanionic clusters [24]. Sodium and calcium atoms are shown in blue; larger and smaller white spheres represent oxygen and hydrogen atoms, respectively. (From Kampf *et al*. [24], used with permission from the *Canadian Journal of Mineralogy and Petrology*.)

Among the most remarkable heteropolyanions are examples of the [(As^3+^V^4+,5+^_12_As^5+^_6_)O_48_] cluster (figure 4*a*), which is found in several rare minerals, including vanarsite, packratite, gatewayite and morrisonite [24]. Consider vanarsite [NaCa_12_(As^3+^V_12_As^5+^_6_O_51_)_2_·78H_2_O], in which [(As^3+^V^4+,5+^_12_As^5+^_6_)O_48_] heteropolyanion clusters are linked to each other by weaker (Na–O), (Ca–O), and hydrogen bonds. In calculating the molecular assembly index for this cluster, we treat the two different oxidation states of arsenic separately because they play distinct structural roles. As in the examples of tourmaline and eudialyte, we take the trigonal symmetry into account, thus first assembling the [As^5+^_2_V_4_O_16_] subcluster, which has a pair of (As^5+^O_4_) tetrahedra, a triad of edge-sharing (VO_6_) octahedra, and a corner-sharing (VO_6_) octahedron (figure 4*a*). Begin by assembling the polyhedral units:

**Table:** 

(As^5+^–O) × 2 → (As^5+^O_2_)	SBMA = 2 steps	EMA = 1 step
(V–O) × 2 → (VO_2_)	2 steps	1 step
(VO_2_) × 2 → (VO_4_) [V octahedron]	1 step	1 step

Next, combine polyhedra into the [As^5+^_2_V_4_O_16_] subcluster:

**Table:** 

(VO_4_) × 3 → (V_3_O_9_) [V octahedral triad]	3 steps	2 steps
(V_3_O_9_) + (VO_4_) → (V_4_O_12_) [V octahedral assembly]	1 step	1 step
(As^5+^O_2_) × 2 + (V_4_O_13_) → [As^5+^_2_V_4_O_16_]	2 steps	2 steps

Three of these [As^5+^_2_V_4_O_16_] subclusters form the ring-like structure:

**Table:** 

[As^5+^_2_V_4_O_16_] × 3 → [As^5+^_6_V_12_O_48_]	2 steps	2 steps

Assembly is completed in 11 or 16 steps by the addition of a central As^3+^ atom, which forms 3 (As^3+^–O) bonds in a single step:

**Table:** 

[As^5±^_6_V_12_O_48_] + (As^3±^–O) × 3 → [As^3±^As^5±^_6_V_12_O_48_]	3 steps	1 step
TOTAL	SBMA = 16 steps	EMA = 11 steps

Note that in this instance the split-branch method results in a significantly higher index (16 steps) than the exact method (11 steps). More than a dozen additional assembly steps, including the numerous sodium, calcium, and hydrogen atoms of the so-called ‘interstitial structure’, are required to complete the crystal structure of vanarsite (figure 4*b*). Additionally, several apical oxygen atoms on both (As^5+^O_4_) tetrahedra and (V^4+^O_6_) octahedra are terminated by (OH^−^) groups—bonds that are not included according to assembly theory protocols.

### 2.5. Paddlewheelite

Heteropolyanions that spontaneously assemble in aqueous solution have been extensively studied in the laboratory [16–18,24]. More than 40 minerals have been described that contain heteropolyanions, which preserve complex signatures of geochemical conditions of formation [16,25]. Often natural heteropolyanions are highly complex [19,20,25], and thus minerals incorporating these clusters represent an excellent opportunity to test hypotheses related to the maximum possible molecular assembly indices for natural inorganic molecules.

**Figure 5.**
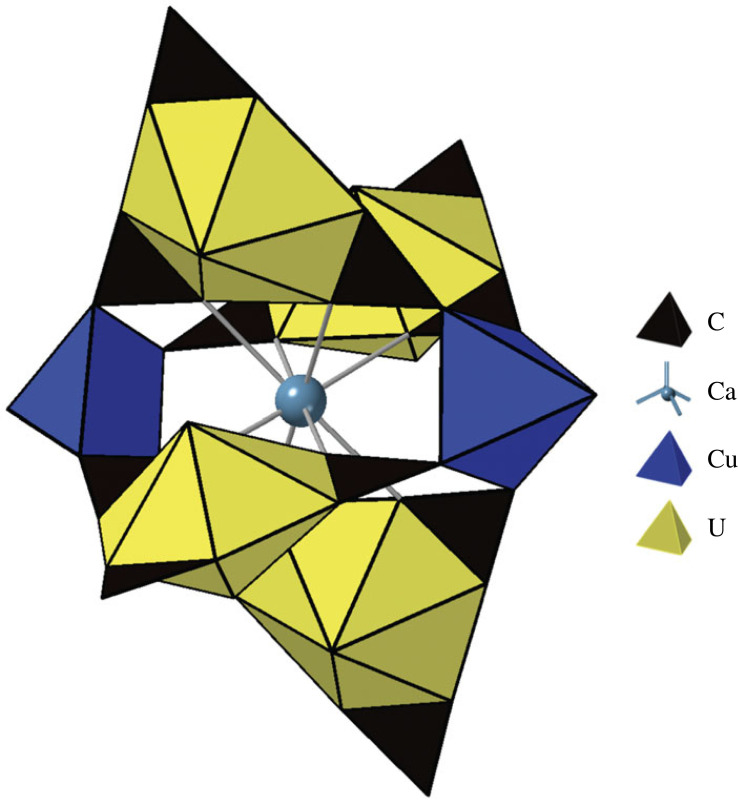
Polyhedral representation of the [CuCa_2_U^6+^_4_O_12_(CO_3_)_12_]^18−^ polyoxometalate subunit, which is a central feature of the complex structure of paddlewheelite [MgCa_5_Cu_2_(UO_2_)_4_(CO_3_)_12_(H_2_O)_33_]. After Olds *et al*. [19].

A variety of complex heteropolyanions occur in secondary uranium-bearing minerals. One fascinating example is the magnesium-calcium-copper-uranyl carbonate mineral paddlewheelite [MgCa_5_Cu_2_(UO_2_)_4_(CO_3_)_12_(H_2_O)_33_] [19], which gets its colourful name from a unique polyanion [(Cu^2+^O)_2_Ca^2+^U^6+^_4_O_10_(CO_3_)_12_]^18−^—a cluster with a (CaCu_2_) ‘axle’ and four paddle-like radiating [(UO_2_)(CO_3_)_3_] groups (figure 5). Assembly of this complex cluster begins with sub-assembly of one of the [(UO_2_)(CO_3_)_3_] planar groups as follows:

**Table:** 

(C–O) × 3 → (CO_3_)	SBMA = 3 steps	EMA = 2 steps
(U–O) × 2 → (UO_2_)	2 steps	1 step
(UO_2_) × 2 → (UO_4_)	1 step	1 step
(UO_4_) + (CO_3_) → [(UO_2_)(CO_3_)]	1 step	1 step

In the above steps, recall that we add bonds, not atoms; therefore, for example, 2 oxygen atoms are shared by U and C in the last of these steps.

**Table:** 

[(UO_2_)(CO_3_)] + (CO_3_) → [(UO_2_)(CO_3_)_2_]	1 step	1 step
[(UO_2_)(CO_3_)_2_] + (CO_3_) → [(UO_2_)(CO_3_)_3_]	1 step	1 step

The next steps add 2 (Ca–O) and a (Cu–O_2_) bond to the paddlewheels:

**Table:** 

(Ca–O) × 2 + [(UO_2_)(CO_3_)_3_] → {Ca[(UO_2_)(CO_3_)_3_]}	3 steps	2 steps
(Cu–O) × 2 → (CuO_2_) [add an apical oxygen to Cu]	2 steps	1 step
(CuO_2_) + {Ca[(UO_2_)(CO_3_)_3_]} → {(CuO)Ca[(UO_2_)(CO_3_)_3_]}	1 step	1 step

Finally, the cluster is completed by combining 4 paddlewheels in 2 steps:

**Table:** 

{(CuO)Ca[(UO_2_)(CO_3_)_3_]} × 2 → {(CuO)Ca[(UO_2_)(CO_3_)_3_]_2_}	1 step	1 step
{(CuO)Ca[(UO_2_)(CO_3_)_3_]_2_} × 2 → [(CuO)_2_CaU_4_O_10_(CO_3_)_12_]	1 step	1 step
TOTAL	SBMA = 17 steps	EMA = 13 steps

The resulting molecular assembly index for paddlewheelite is 13 or 17, depending on the method used.

### 2.6. Ewingite

**Figure 6.**
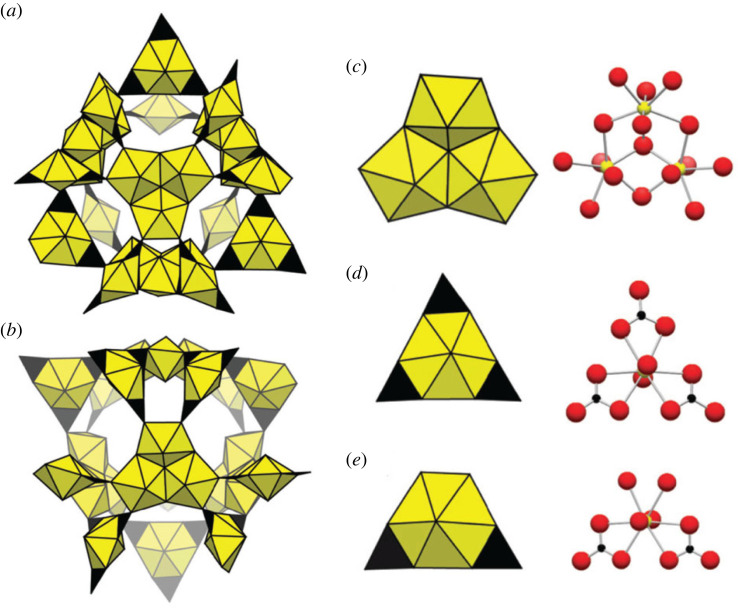
Portions of the complex ewingite structure [Mg_8_Ca_8_(UO_2_)_24_(CO_3_)_30_O_4_(OH)_12_(H_2_O)_138_], showing polyhedral clusters (from [20]). (*a*,*b*) Polyhedral representations of the uranyl carbonate cage in two different orientations. (*c*) Both polyhedral and ball-and-stick representations of the ‘fundamental building unit’ FBU-1, with (*d*) FBU-2 and (*e*) FBU-3. Uranyl pentagonal and hexagonal bipyramids are yellow, carbonate triangles are black. Uranium, carbon, and oxygen atoms are shown as yellow, black, and red spheres, respectively. Not shown in this figure are numerous connecting Mg and Ca polyhedra, as well as (OH) groups.

The most complex mineral structure identified thus far is the hydrous magnesium-calcium-uranyl carbonate, ewingite [Mg_8_Ca_8_(UO_2_)_24_(CO_3_)_30_O_4_(OH)_12_(H_2_O)_138_], with only six different essential elements but a remarkable 121 different atomic sites in its asymmetric unit [20]. Embedded in ewingite is a molecule-like uranyl carbonate ‘cage’ with composition [(UO_2_)_24_(CO_3_)_30_O_4_]^20−^, which in turn is made from three different polyhedral building units: four of [U_3_O_16_], six of [(UO_4_)(CO_3_)_2_], and six of [(UO_2_)(CO_3_)_3_] (figure 6). In spite of the overall structural complexity, the assembly of the molecular subunit is simplified by the numerous repeating (U–O) and (C–O) bonds in (UO_7_), (UO_8_), and (CO_3_) polyhedra, as well as its pseudo-trigonal symmetry.

Consider first the (U_3_O_16_) cluster (figure 6*c*), which requires a triad of edge-sharing (UO_7_) polyhedra, which can be assembled by combining one unit with 6 (U–O) bonds to 2 units with 5 (U–O) bonds.

**Table:** 

(U–O) × 2 → (UO_2_)	SBMA = 2 steps	EMA = 1 step
(UO_2_) + (U–O) → [UO_3_]	1 step	1 step
(UO_3_) + (UO_2_) → (UO_5_)	1 step	1 step
(UO_3_) × 2 → (UO_6_)	1 step	1 step

Thus, 4 steps yield the basic uranium coordination groups required for cluster 1 (figure 6*c*), which is further assembled by combination:

**Table:** 

(UO_5_) × 2 + (UO_6_) → (U_3_O_16_)	2 steps	2 steps

Four additional steps are required to assemble cluster 3, [(UO_4_)(CO_3_)_2_] (figure 6*e*), which incorporates a (UO_8_) polyhedron with two peripheral (CO_3_) groups:

**Table:** 

(UO_6_) + (UO_2_) → (UO_8_)	1 step	1 step
(C–O) × 2 → (CO_2_)	2 steps	1 step
(UO_8_) + (CO_2_) × 2 → [(UO_4_)(CO_3_)_2_]	2 steps	2 steps

Only one additional step is required to assemble cluster 2, [(UO_4_)(CO_3_)_3_] (figure 6*d*), which adds a third peripheral (CO_3_) group to cluster 3:

**Table:** 

[(UO_4_)(CO_3_)_2_] + (CO_2_) → [(UO_4_)(CO_3_)_3_]	1 step	1 step

Note that several alternative assembly pathways might be employed to form clusters 1, 2, and 3 from the constituent bonds, but all pathways appear to require a minimum of 11 assembly steps.

Assembly of the entire cluster requires attaching one or two [(UO_4_)(CO_3_)_2_] and [(UO_2_)(CO_3_)_3_] groups to each of four [U_3_O_16_] subclusters (4 steps):

**Table:** 

[(UO_4_)(CO_3_)_2_] + [(UO_2_)(CO_3_)_3_] → [(UO_2_)_2_(CO_3_)_5_]	1 step	1 step
[(UO_2_)_2_(CO_3_)_5_] + [(UO_4_)(CO_3_)_2_] → [(UO_2_)_3_(CO_3_)_7_]	1 step	1 step
[(UO_2_)_3_(CO_3_)_5_] + [(UO_2_)(CO_3_)_3_] → [(UO_2_)_3_(CO_3_)_8_]	1 step	1 step
[(UO_2_)_3_(CO_3_)_7_] + [U_3_O_16_] → [(UO_2_)_6_(CO_3_)_7_O]	1 step	1 step
[(UO_2_)_3_(CO_3_)_8_] + [U_3_O_16_] → [(UO_2_)_6_(CO_3_)_8_O]	1 step	1 step

The final assembly steps involve combining 2 [(UO_2_)_6_(CO_3_)_7_O] and 2 [(UO_2_)_6_(CO_3_)_8_O]:

**Table:** 

[(UO_2_)_6_(CO_3_)_7_O] + [(UO_2_)_6_(CO_3_)_8_O] → [(UO_2_)_12_(CO_3_)_15_O_2_]	1 step	1 step
[(UO_2_)_12_(CO_3_)_15_O_2_] × 2 → [(UO_2_)_24_(CO_3_)_30_O_4_]	1 step	1 step
TOTAL	SBMA = 20 steps	EMA = 18 steps

Thus, it requires a total of 18 or 20 steps to assemble the [(UO_2_)_24_(CO_3_)_30_O_4_] heteropolyanion.

The structure of ewingite was difficult to determine and refine owing to the quality of the available crystals. Synthetic analogues similar to this cluster have since been reported [26,27], but containing four [U_3_O_16_] subclusters and 12 [(UO_2_)(CO_3_)_3_] subclusters. It is possible that the cluster of ewingite also contains 12 [(UO_2_)(CO_3_)_3_], as opposed to 6 each of [(UO_2_)(CO_3_)_3_] and [(UO_2_)(CO_3_)_2_]; however, the diffraction data failed to reveal the locations of disordered carbonate groups. In the event that this is the case, a minimum of 16 steps would be needed to assemble the ewingite cluster.

### 2.7. Ilmajokite

**Figure 7.**
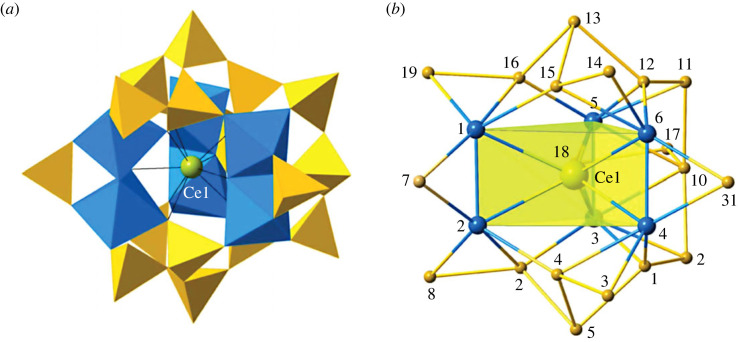
The complex structure of ilmajokite [Na_11_KBaCe_2_Ti_12_Si_37.5_O_94_(OH)_30_·29H_2_O] (adapted from [28]) incorporates trigonal prismatic titanosilicate clusters of composition [Ce(Ti_2_SiO_12_)_3_(Si_17_O_31_)], which is possibly the most complex known molecule-like subunit thus far observed in minerals. Titanium octahedra (blue) and silicon tetrahedra (yellow) form a cage-like structure around a central cerium atom (green sphere). Note that this cluster model does not include numerous apical OH^−^ (i.e. silanol) groups on silicate tetrahedra.

Ilmajokite [Na_11_KBaCe_2_Ti_12_Si_37.5_O_94_(OH)_30_·29H_2_O] is a cerium-bearing titanium silicate of exceptional structural complexity, with 236 unique atom positions in the asymmetric unit [28]. The monoclinic structure incorporates a trigonal prismatic titanosilicate (TPTS) cluster of composition [Ce(Ti_2_SiO_12_)_3_(Si_17_O_35_)], which is among the most complex known molecule-like entities thus far observed in minerals (figure 7*a*,*b*). The core of each cluster consists of three separate (Ti_2_O_10_) dimers of edge-sharing titanium octahedra that create a nine-coordinated site for one central Ce^3+^ ion—hence a [Ce(Ti_2_O_10_)_3_] group. Each (Ti_2_O_10_) group is also bonded to individual (SiO_4_) tetrahedra (including tetrahedra numbered 7 and 31 in figure 7*b*), resulting in a [Ce(Ti_2_SiO_12_)_3_] subassembly. The periphery of this group is decorated by 17 corner-linked silicate tetrahedra in a complexly branched (Si_17_O_52_) chain that wraps around all six titanium octahedra (including 17 corner-shared O atoms as attachment points). The structure of this tetrahedral assembly (figure 7*b*) can be described by a linear 11-tetrahedron chain (beginning with tetrahedron 8 (here abbreviated ‘T8’; figure 7*b*) and ending at T19), with T3 and T4 branching off T5; T14 and T15 branching off T13; and T17 and T18 branching off T10. This topology is analogous to that of 3,6,9-ethylundecane, but with corner-linked silicate tetrahedra instead of single-bonded carbon atoms.

Calculation of the molecular assembly index of this 98-atom cluster begins with the central [Ce(Ti_2_SiO_12_)_3_] subassembly, which has pseudo-trigonal symmetry:

**Table:** 

(Ti–O) × 2 → (TiO_2_)	SBMA = 2 steps	EMA = 1 step
(TiO_2_) × 2 → (TiO_4_)	1 step	1 step
(TiO_4_) + (Ti–O) → (TiO_5_)	1 step	1 step
(TiO_5_) × 2 → (Ti_2_O_10_)	1 step	1 step

Each of 3 (Ti_2_O_10_) dimers must be bound to an apical (SiO_4_) tetrahedron, as well as the central (CeO_9_) polyhedron:

**Table:** 

(Si–O) × 2 → (SiO_2_)	2 steps	1 step
(SiO_2_) + (Ti_2_O_10_) → (Ti_2_SiO_12_)	1 step	1 step
(Ce–O) + (Ti_2_SiO_12_) → [Ce(Ti_2_SiO_12_)]	2 steps	1 step
[Ce(Ti_2_SiO_12_)] + (Ti_2_SiO_12_) → [Ce(Ti_2_SiO_12_)_2_]	1 step	1 step
[Ce(Ti_2_SiO_12_)_2_] + (Ti_2_SiO_12_) → [Ce(Ti_2_SiO_12_)_3_]	1 step	1 step

Thus, a total of 9 or 12 assembly steps is required for the central [Ce(Ti_2_SiO_12_)_3_] subunit. Assembly of the branched (Si_17_O_52_) silicate tetrahedral chain proceeds as follows, noting that (SiO_2_) has already been assembled above (refer to figure 7*b*):

**Table:** 

(Si–O) + (SiO_2_) → (SiO_3_) [construction of T8]	1 step	1 step
(SiO_3_) × 2 → (Si_2_O_6_) [linking T8 to T2]	1 step	1 step
(Si_2_O_6_) × 2 → (Si_4_O_12_) [linking T8-T2 to T5-T4]	1 step	1 step
(Si_4_O_12_) + (SiO_3_) → (Si_5_O_15_) [T8 to T3]	1 step	1 step

This is a chain with 5 tetrahedra that repeats 3 times between T8 and T14:

**Table:** 

(Si_5_O_15_) × 3 → (Si_15_O_45_) [tetrahedral chain from T8 to T14]	2 steps	2 steps

To complete the silicate subcluster, add T16 and T19 (i.e. Si_2_O_7_) to T13 of the previous assembly:

**Table:** 

(Si_2_O_6_) + (Si–O) → (Si_2_O_7_)	1 step	1 step
(Si_15_O_45_) + (Si_2_O_7_) → (Si_17_O_52_)	1 step	1 step

Thus, the branched silicate chain requires 8 steps. The final assembly step is to attach the branched silicate chain to the central [Ce(Ti_2_SiO_12_)_3_] cluster, requiring 1 additional step:

**Table:** 

(Si_17_O_52_) + [Ce(Ti_2_SiO_12_)_3_] → [Ce(Ti_2_SiO_12_)_3_ (Si_17_O_35_)]	1 step	1 step
TOTAL	SBMA = 21 steps	EMA = 18 steps

Consequently, the molecular assembly index of the ilmajokite heteropolyanion is 18 or 21, depending on the method used.

## 3. Mineral structural complexity and assembly indices

Krivovichev *et al.* [21,29–34] have introduced a number of metrics to characterize the chemical and structural complexity of minerals. Of greatest relevance to this study are calculations of the structural complexity per unit cell (in bits). Krivovichev *et al*. [34] recorded structural complexity values for 4596 mineral species—approximately 75% of all IMA-approved mineral species.

Structural complexity is estimated as the amount of structural Shannon information per atom (^str^*I*_G_) and per unit cell (^str^*I*_G,total_) calculated according to the following equations [21,29]:

3.1 $${\;}^{\mathrm{str}}I_{G}=\text{–}\sum_{i=1}^{k}\;p_{i}\log_{2}p_{i}\left(\frac{\mathrm{bit}}{\mathrm{atom}}\right)$$

and

3.2 $${\;}^{{\;}^{\mathrm{str}}}I_{G,\mathrm{total}}=v\times^{{\;}^{\mathrm{str}}}I_{G}=\text{–}v\times \sum_{i=1}^{k}\log_{2}p_{i}\left(\frac{\mathrm{bit}}{\mathrm{cell}}\right),$$

where *k* is the number of different crystallographic sites in the structure and *p_i_* is the random choice probability for an atom from the *i*th crystallographic site, that is:

3.3 $$p_{i}=\frac{m_{i}}{v},$$

where *m_i_* is a multiplicity of a crystallographic orbit (i.e. the number of atoms of a specific Wyckoff site in the reduced unit cell) and *v* is the total number of atoms in the reduced unit cell. The resulting values of structural complexity per unit cell range from zero for 48 native element mineral species that have no degrees of freedom (e.g. diamond), to the most complex structure known, ewingite with 23 478 bits per unit cell.

Mineral assembly indices and complexity per unit cell differ significantly in their derivations; however, as might be expected the two measures display some correlations. The most complex crystal structures also tend to have the highest assembly indices.

In their list of the 20 most complex known mineral structures, all with more than 3000 bits per unit cell, Krivovichev *et al*. [33] reveal at least six characteristics that lead to complexity:

(1) the presence of large polyoxometalate clusters, often exceeding a nanometre in diameter;
(2) the presence of large clusters linked to form an even larger framework topology;
(3) three-dimensional modular frameworks that incorporate multiple cage-like topologies;
(4) formation of modular layers that can combine in multiple topologies;
(5) high-hydration states in salts with complex heteropolyhedral units; and
(6) formation of ordered superstructures of relatively simple structure types.

Most of these conditions lead to a high number of different local coordination topologies—factors that appear to be representative of minerals with molecule-like substructures with MA index ≥10 (table 1). Only in the latter instance (criterion 6) of superstructures based on simple subunits, with the same coordination states appearing over and over again, will the assembly indices be low compared to the structural complexity.

A survey of all 4596 known mineral structures [33,34] reveals that 1600 minerals have relatively simple structures with ≤100 bits per unit cell—a value typical for minerals with an assembly index ≤6 (table 1). Another approximately 1600 minerals have between 100 and 500 bits per unit cell, typically corresponding to crystalline phases with assembly indices up to 10 in our study. By contrast, only about 750 of the 4596 minerals surveyed (16%) of all minerals have >500 bits per unit cell, some of which correspond to phases in our calculations with assembly indices >10. Of these more complex minerals, only 369 of 4596 (8%) display >1000 bits per unit cell—most of which have assembly indices significantly greater than 10.

It is important to note that of these most complex minerals, a significant number, including paddlewheelite, ewingite and vanarsite, arise as secondary oxidized and hydrated phases of precursor primary phases. Therefore, it might be argued that biology (principally owing to atmospheric oxygenation via photosynthesis) played a role in their formation [23]. However, in theory, these oxidized minerals could form on lifeless terrestrial worlds whose near-surface environments have been oxidized via photochemical processes and atmospheric escape of hydrogen [35–37]; thus, their formation may not unambiguously reflect the presence of a biosphere. Importantly, other minerals with complex molecular substructures, such as ilmajokite and members of the eudialyte, vesuvianite and tourmaline groups, form exclusively as primary igneous or metamorphic phases—i.e. with no biological influence.

## 4. Conclusion

We draw several conclusions from this investigation of the MA indices of naturally occurring inorganic molecules:

— Some naturally occurring heteropolyanion clusters, including those in ewingite and ilmajokite, have assembly indices greater than the proposed abiotic/biotic cutoff of 15, thus invalidating the claim that only biological processes can produce molecules with assembly indices ≥15.
— Heteropolyanion clusters of complexity greater than those of ewingite and ilmajokite likely occur in nature. All of the 5 most complex structures listed in table 1 were determined since 2016. The structures of more than 1000 known minerals, many of them extremely complex, have yet to be fully refined [34]. Comparable examples of natural complex heteropolyanions with molecular assembly indices exceeding 14 steps for strongly bonded molecular subunits of mineral structures are possibly among the minerals containing heteropolyanions reviewed by Krivovichev [25].
— Hystad and colleagues [38] have demonstrated that more than 3000 additional mineral species on Earth exist, but have yet to be discovered and described. New mineral species are being discovered at a rate of approximately 100 species per year [39], and many of those display complex, previously unknown structures [34,40].

A principal objective of this study is to test the hypothesis of Marshall *et al*., who suggest that ‘There is a critical value of pathway complexity above which all artefacts must be biologically derived’ [1], while ‘complex molecules found in high abundance are universal biosignatures’ [3]. They suggest that only living cells generate significant concentrations of structures with MA index ≥15. Our evaluation of this central thesis of assembly theory is direct and quantitative. The present study of mineral assembly indices reveals that, while most common molecule-like structures in minerals have MA indices ≤ 10, the most complex mineral species have molecule-like subunits with MA indices ≥ 21. What does this result imply in the context of the search for agnostic biosignatures? One of two conclusions might be drawn.

First, it is possible that the complex molecular heteropolyanions incorporated into mineral crystals are fundamentally different from organic molecules in their assembly rules. Therefore, the fact that some inorganic minerals have molecule-like subunits that can achieve assembly indices greater than 15 might be irrelevant in the search for life. In this case, the rhetoric associated with molecular assembly theory should be clarified to exclude exclusively inorganic species. However, such an exclusion would seem to violate the very premise of assembly theory's claims regarding unambiguous biosignatures based on molecular complexity.

Alternatively, given the plausibility of the assembly theory contention that only life can produce multiple copies of extremely complex objects, it is possible that a value (or range of values) of assembly index greater than 15 may represent a more realistic, if ‘fuzzy’, divide between abiotic and biotic structures. In this regard, an intriguing topic for speculation relates to the potential for ‘open-ended evolution’ of different natural systems [41–43]. We suggest that minerals display bounded evolution, with the implication that there exists an upper limit to the molecular assembly index for naturally occurring heteropolyanions—perhaps a limit not much greater than 21. Life, by contrast, may display unbounded evolution, with the possibility that objects with ever-increasing assembly indices may emerge, initially through biological evolution and subsequently through technological evolution.

In either case, we conclude that significant structural complexity of molecules is not the unique province of biochemistry and that natural inorganic chemistry has the potential to generate significant local populations of molecular structures with MA indices greater than 15. In that case, the possibility should be entertained that abiotic processes might also produce *organic* crystals of great complexity on a carbon-rich, abiotic planet or moon, especially given billions of years of abiotic organic mineral evolution in the absence of life. For example, Maynard-Casely *et al*. [44, table 2] and Cable *et al*. [45, table 1] have proposed the existence of complex organic co-crystals on Titan. Examples of these compounds, all of which reflect laboratory synthesis under plausible Titan conditions, include the bimolecular co-crystals (1:4) 1,3-butadiene:urea and (1:1) acetonitrile:3,4-dihydroxybenzoic acid, as well as the tri-molecular co-crystals (1:1:1) acetonitrile:cyclohexane-1,3-*cis*,5-*cis*-tricarboxylic acid and (1:2:1) acetonitrile:4-hydroxybenzoic acid:2,3,5,6-tetramethylpyrazine. The estimated molecular assembly indices of these proposed Titan organic minerals range from 5 to 10. If such abiotic compounds do exist on Titan, then, by analogy with the multi-stage congruent evolution of minerals on Earth [46,47], local pockets of much greater abiotic molecular complexity are likely to persist, as well.

An important conclusion of prior studies is that planets and moons, given sufficient time and chemical processing, display remarkable degrees of mineral evolution, which include striking increases in the average diversity and complexity of minerals [33,34,46,47]. On Earth, the earliest minerals formed by high-temperature condensation display an average complexity per unit cell of approximately 83 bits, with no minerals incorporating molecule-like subunits with MA indices >6. Following the advent of plate tectonics, that average structural complexity value increased to 275 bits per unit cell, with tourmaline and eudialyte group minerals displaying molecule-like subunits with MA indices ≥10. By contrast, the average complexity of mineral species formed in tandem with today's terrestrial biosphere exceeds 370 bits per unit cell, with MA indices of some heteropolyanionic clusters significantly exceeding 15. These trends suggest that planets and moons can generate chemical compounds of significant structural complexity through purely abiotic processes. Furthermore, the processes leading to mineral complexification are congruent—i.e. each stage of mineral evolution provides new phases that have the potential for further complexification [46–48].

We have demonstrated that abiotic chemical processes have the potential to form crystal structures of great complexity—values exceeding the proposed abiotic/biotic divide of MA index = 15 [3]. In this context of dynamic, evolving planetary environments, the absence of concentrations of complex abiotic organic molecules on Earth is not surprising. The ubiquity of complex cellular life would certainly mask an abiotic suite of complex molecules, assuming that such molecules had not been long since consumed by a voracious biosphere. However, no such constraints would seem to affect Titan and other organic-rich abiotic worlds, where complex molecular species in the form of co-crystals may abound.

In conclusion, while the proposal of a biosignature based on a molecular assembly index of 15 is an intriguing and testable concept, the contention that only life can generate molecular structures with MA index ≥ 15 is in error. Furthermore, in spite of amusing speculations in the literature of science fiction [49], it is unlikely that the definition of any universal phenomenon as complex and diverse as ‘life’ can be reduced to a scalar.

## Data Availability

This article has no additional data.
